# Mr. Sheldrake, in Reply to Mr. Watt

**Published:** 1801-03

**Authors:** T. Sheldrake

**Affiliations:** No. 50, Strand


### *To the Editors of the Medical and Phyfical Journal.*

GENTLEMEN,

IN declaring my refolution, through the medium of your Journal, not to anfwer your correfpondent, Mr. Watt, I was actuated by motives of refpect towards yourfelves, and decorum to your readers; as, I conceive, your object is to promulgate the knowledge of truth, and theirs to acquire ufeful information; neither of which can be promoted by the continuance of a difpute, which, by the conduct of my opponent, has degenerated into PERSONAL ABUSE; fo thinking, and acting from fuch motives, it is not without furprize that I perceive you have printed in your Journal for the prefent month, a letter from him, in which I am charged with having declined the controverfy, becaufe I find *my attack no longer defenfible, and therefore get rid of it in the beft way I can*; and likewife, am loaded with fome more of that kind of perfonality which has has already placed him beneath my farther notice. It is not for me to queſtion your motives for inſerting his letter, as I have no doubt that that juſtice which regulates your conduct, will induce you to publiſh this anſwer, which *that letter has made indispensably necessary.*

He ſays, "he leaves your candid readers to determine, whether my anſwer to his *original* communication, or his reply, contains moſt unprovoked, illiberal, and PERSONAL ABUSE." If they have not yet determined this point, they certainly will do ſo; and, perhaps, the following recapitulation may facilitate their labour in making the deciſion.

Mr. W. communicated to you what he was willing to make your readers believe was an *invention or discovery* of his own. Without attempting to inveſtigate his own actual and private knowledge on this point, I ſhall be permitted to obſerve, that every man who makes his own diſcoveries public, hopes to gain reputation, at leaſt, by the communication; and every Plagiary who meanly extracts from the works of others, any thing which he artfully attempts to introduce as his own, hopes to purloin that reputation to which his conſcience tells him he has no right. Mr. W. ſtands in one of thoſe ſituations; and whichever it may be, it was natural for him to loſe his temper, when he found himſelf diſappointed of the objects he had in view.

Every man who writes, has an undoubted right to arrange his ſubject in that manner which appears to him to be the beſt; I heartily pity the man, who ſits deliberately down to write all that he can utter upon any ſubject. I was willing to make my anſwer as brief as the nature of the caſe would allow, and, therefore, only mentioned ſuch circumſtances as were matters of great notoriety in this part of the world; I enlarged on ſuch as required elucidation, and declared my intention to reſume the ſubject at a future opportunity: Perhaps, moſt of your readers underſtood, that if any thing mentioned in that anſwer required explanation, it would be explained when the ſubject was reſumed; perhaps Mr. W. FEARED this might be the caſe; and if he did not ſeriouſly intend to put an end to a diſcuſſion, from which he could expect no gratification, he *accidentally* hit upon a method of doing ſo moſt effectually.

In anſwering Mr. W. I ſaid nothing *directly*, or by implication OF HIM, either as an individual or as a profeſſional man, for the beſt of all reaſons,—I knew nothing about him: But of his work, as it appeared in your Journal, I was able to judge, and even thought myſelf capable of enabling thoſe to form a correct opinion of it, who had not reflected ſo much upon it as I have done; and your readers will determine whether I have or have not ſucceeded: At leaſt, they will allow there was nothing improper in the attempt; the work was a fair object of criticifm; and if its author had afferted things that were not true, if he had exaggerated facts, and drawn conclufions that did not regularly follow from the premifes laid down, is he to blame *himfelf* for the unenviable fituation in which he has placed himfelf; or *me*, for fhewing the exact quantity of refpect his communication is entitled to?

Mr. W. had an undoubted right to reply to my anfwer, and I believe he made the beft anfwer his talents would enable, and his temper would permit him to make; but *fearing* that it would not effectually convince his readers, or filence me, he called in an auxiliary, which, like many others, can only difgrace the caufe it is employed in: He turned *from* the fubject *to* the author, and applied epithets to me which I could only anfwer properly, by treating with filent contempt.

As this is the act which juftifies me in faying he is fallen too low for me to notice, the following quotations are produced in proof. P. 499. "I fhould certainly have fufpected, had Mr. S. been writing another *Practical Effay* on diftorted Limbs, that he had foifted in the *Shoe-maker* with little other motive than merely to *fwell* the fize of his volume."

Again, "Indeed, with regard to the labours of Mr. Holmes, *the Shoe-maker, and with him I may* ASSOCIATE *Mr. Sheldrake, the Trufs-maker,*" *&c.*

Again, p. 502. "Let him have recourfe to the laws of his nation, and feek redrefs in its proper channel, and not with his NATIVE EFFRONTERY, *criminate* thofe by BARE ASSERTIONS, whom he cannot *condemn* by SOUND ARGUMENTS.

If I underftand the conftruction of language, the firft is a direct infinuation, that I am capable of fabricating a book, by foifting in matter unconnected with the fubject, merely *to swell the fize of the volume*; in other words, endeavouring to obtain money by falfe pretences. Of the fecond, I am to underftand, that, I had defcribed Holmes, * the Shoe-maker, as an ignorant perfon, who had pretended to do what he was unable

* As this man has been dead many years, he can fuftain no injury by his name being thus publicly mentioned. It is, indeed, neceffary to do this, as it affords a fingular proof of Mr. W's accuracy, and will fhew how incapable he is, of diftorting any thing to anfwer his own purpofes. I mentioned that Holmes had been dead feveral years; Mr. W. made what obfervations he thought proper on the paffage, and then proceeds, "*If there* IS *fuch a man as Mr. Holmes, &c.*" If any one fhould inveftigate the fubject, to fhew Mr. W's fide of it, he would firft enquire for Mr. H. and finding there IS no fuch perfon, his argument would run thus, "AS THERE IS *no fuch perfon, &c.*" and he would form many conclufions that would be highly favourable to Mr. W's views.

able to perform; Mr. Watt, by *associating* me with him in *Italics*, meant to insinuate, that I was a man of the same description. The third is too plain to require explanation, and is that kind of charge, which no man would make in the face of another, and which Mr. W. has made very courageously, when at a distance that secures him from all the consequences. This part of Mr. W's reply, I call *slanderous personal abuse*.

This Mr. W. affects to consider as a victory; it is a singular one, however, and considered as a controversial artifice is not without merit. If it can be established as a precedent, there are many men who, like Mr. W. are deficient in argument and temper, and could easily supply the defect by the application of such terms as preclude all reply in writing, and of course, upon this principle, will be victorious in every contest they engage in.

This victory, such as it is, will not be of long duration, for I have promised to convey to you, an account of my method of curing these diseases, which cannot be done without previously examining other theories that have preceded my own. And in the course of that communication, your readers will be enabled to ascertain, whether in my reply to Mr. W. I advanced a single circumstance that deviates from the strictest truth.

Mr. W. seems to be endued with so much of the faculty of *second sight*, as enables him to foresee this; and this foresight is, I believe, the real cause of the notice he favours me with in your last Journal. He had advanced, with all the dogmatism of infallibility, that *all distortions in the legs and feet of children, by whatever cause produced, or* HOWEVER FORMIDABLE *they* MIGHT APPEAR, might be cured by the application of the same means: The two cases I have transmitted to you, prove the reverse of this; for though they are cases of the same disease, they are in circumstances so different, that no one will believe, and even Mr. W. has not attempted to say, that the *same means* that were employed to cure the one, could have cured the other. This is fatal to his dogma, and this is the shaft that rankles in the breast of Mr. W. But though he knows this, he is too uncandid a disputant to confess it. As the doctrine to be deduced from these cases, is directly opposed to his dogma, he would have been right in saying, THEY were directed against *it*; or, if he pleases, against *him*; but he must, at the same time, have admitted his own mistake. This he had not philosophy enough to do, nor had he fortitude sufficient to be silent, he therefore pretends to discover, that in conceiving the use of the terms* varus

* Lest Mr. Watt should imagine, that I decline the discussion of this point too, *because it is not defensible*, I beg leave to observe that the terms varus and valgus, only describe the position of the foot, but convey no ideas respecting varus and valgus, my cenfure is *principally directed against* HIM. Now, as he proves that the fame words have been ufed in the fame fenfe, " not only by medical writers, but even by claffical authors, for at leaft two thoufand years," and as my cenfure was expreffed in the moft general terms, it is moft certain that it could not be directed principally againft HIM, unlefs *he* is the *principal* of all the medical and all the claffical authors who have flourifhed during that time. If the conftruction of my language will not bear me out, I beg leave folemnly to declare, that I meant no reference to Mr. W. in that paffage, though I certainly intended that the *facts* of thofe cafes fhould be oppofed to the *fallacy* of his doctrines. It is whimfical in Mr. W. to fay he " *hates controversy*,* while he fhews, that he poffeffes in perfection one talent of the greateft value to a profeffed controvertialift, viz. that of evading thofe parts of a fubject on which he is completely defeated, and fubftituting others from which he expects to derive lefs difgrace. Of this talent, the paffage abovementioned is one compleat fpecimen; another exifts in his reply to my anfwer; this it becomes me to notice, as this is the only opportunity I fhall have of doing fo, and as it is intimately connected with that part in which he fo elegantly mentions my *native effrontery*.

In his original communication, he fays, " When I firft thought of this inftrument, I had no idea of its being applied to any other cafe but the *vari & valgi*; and there only when the diforder happened to lie in the ancle joint; but *I have since*

---

refpecting the ftate of the parts concerned in the difeafe. The term Club-foot is, perhaps, a vulgarifm, poffibly a local term, but certainly conveys no correct idea beyond that of a diftorted foot; if, then, I fay fuch a patient has a Club-foot, and add an accurate defcription of the ftate of the parts concerned in the difeafe, and give the beft information that the nature of the cafe will allow, and that a profeffional man can require, for from the *whole of the defcription* he underftands the real fituation of the patient; but if I fhould only fay, he has a varus, or valgus; and if I fhould quote Plautus, to prove that thefe were *good old claffical Latin terms*, I fhould obtain more reputation for learning than for capacity to make others underftand the nature of the difeafe I meant to defcribe.

* When on a former occafion, Mr. W. found that part of his writing bore a different interpretation from what he intended, he made a fingular attempt to efcape the dilemma, by obferving, that *either* the word NOT was improperly inferted in the manufcript, OR, that it had been interpolated by the Printer. I made an experiment, by omitting the word *not*, but the fenfe was not improved by the omiffion, therefore the excufe could not ferve him; but on the prefent occafion, it may do extremely well, as I think it very probable that Mr. W. wrote, " *I hate contradiction*," which the compofitor has, by miftake, fet up, " *I hate controverfy*;" this amendment will not only improve the reading, but will likewife prove the fact.

*ſince thought*, that by a ſmall alteration it may be applied to all the different kinds of diſtorted limbs with conſiderable eaſe and advantage."

He then deſcribes his *notion* of applying a ſpring, to cure curvature of bones of the leg, and refers to a figure in his plate. It is this notion, or idea, which I believe to have been borrowed, copied, or by whatever term plagiariſm may be deſigned, from the ſpecification of my patent To prove this, I cited the paſſage at large from the ſpecification, compared it with the paſſage in Mr. W. and by referring to the figures in the plates, it will appear, that the diagram by which I illuſtrate the principle, exactly reſembles that application of a ſpring which Mr. W. calls his own invention.

The inference is *inevitable*; the reſemblance between the two paſſages is compleat. The priority of publication is with me; *therefore*, it is moſt certain, *either* that by a ſtrange coincidence of circumſtances, Mr. W. did invent that which I had publiſhed ſome years before, *without the leaſt knowledge of my publication*, or that he had taken from the latter an idea, which he had tranſplanted into his own, and through the medium of your Journal offered it to the public as an *idea of his own:* Thus meanly ſeeking to deprive me of what reputation may attach itſelf to the diſcovery.

Whatever doubt then exiſted as to the nature of the fact, has been diſſipated by Mr. Watt; he knows the operation of his own mind on the ſubject; and if this idea had originated with himſelf, he could have eaſily ſhown that it did ſo, for the exiſtence of ſimilar paſſages in different authors does not neceſſarily imply plagiariſm in either. Has Mr. Watt done this? No! With the ſpirit of a freebooter, who knows himſelf to be out of the reach of the law,* he exclaims, "*If my inſtrument nearly reſembles Mr. Sheldrake's, it is equally efficacious*; if more imperfect, it need not have given him any alarm; and inſinuates, that I fear him as a rival: And aſks, in a tone of bravado, why I do not have recourſe to the law?

To obviate all the miſrepreſentation that Mr. W. is evidently willing to derive from this ſource, I beg leave to obſerve, that according to my theory of theſe diſeaſes, and the treatment I have

* This ſimile may ſound harſhly, but it is moſt ſtrictly juſt; the protection of property by patent granted in England, does not extend to Scotland; *therefore*, if Mr. Watt had copied fifty patent inventions, for which the law might puniſh him in this country, he might enjoy the fruits of his ingenuity in his own in perfect ſafety, for *our laws could not reach him there*; of courſe, his aſking why I did not have recourſe to the laws of *my* country, is mere vapour.

I have founded upon it, not only no one kind of inftrument can cure all the varieties of the difeafe, but no one fpecimen of the difeafe can be cured by the fame inftrument; this will be fully explained hereafter: In the mean time, I beg leave to obferve, that if a congrefs was affembled, of all the patients who have been under my care, with their friends, and the medical men who have attended them, they would unanimoufly determine, that Mr. W. has not at prefent the moft diftant comprehenfion of my plan; that the particular part which he has borrowed from my book, is not what, he fays, he *has* practifed, but what he *thinks*, "may be applied to all the different kinds of diftorted limbs with confiderable eafe and advantage;" and to this I beg leave to add, that though the principle is undoubtedly true, whoever fhall try that particular modification of it which he recommends, will find it cannot anfwer the purpofe he recommends it for.

With this exertion of my *native effrontery*, I fhall take leave of Mr. Watt, unlefs you fhould indulge him, by publifhing more of his perfonal reflections on myfelf: It is to repel *fuch reflections only* that I have written this; and truft, that *you* will fee the propriety of putting an end to *fuch* difputes. But whatever may be advanced on profeffional fubjects ought to be liable to accurate inveftigation, which I fhall never wifh to avoid, and which, I hope, will continue to be the chief object of your valuable Journal.

In my next, I fhall continue the fubject of my laft; I fhould have done fo before, but I am waiting for the opinion of a gentleman who is at a diftance from town, and which I think effential to the confirmation of fome facts I mean to lay before you; as foon as I receive it, they will be communicated. I am, &c.

