# Errata

**Published:** 1801-04

**Authors:** 


### ERRATA.

Page 267, line the laſt, for *conceiving*, read *censuring*.

